# Comparison of four performance models in quantifying the inequality of leaf and fruit size distribution

**DOI:** 10.1002/ece3.11072

**Published:** 2024-03-01

**Authors:** Lin Wang, Ke He, Cang Hui, David A. Ratkowsky, Weihao Yao, Meng Lian, Jinfeng Wang, Peijian Shi

**Affiliations:** ^1^ Department of Applied Mathematics, College of Science Nanjing Forestry University Nanjing China; ^2^ Architectural Design and Research Institute Shenzhen University Shenzhen China; ^3^ Department of Mathematical Sciences, Centre for Invasion Biology Stellenbosch University Stellenbosch South Africa; ^4^ Mathematical and Physical Biosciences, African Institute for Mathematical Sciences Cape Town South Africa; ^5^ Tasmanian Institute of Agriculture University of Tasmania Hobart Tasmania Australia; ^6^ Bamboo Research Institute, College of Ecology and Environment Nanjing Forestry University Nanjing China

**Keywords:** Akaike information criterion, close‐to‐linear behavior, Gini index, goodness of fit, Lorenz curve, parameter‐effects curvature

## Abstract

The inequality in leaf and fruit size distribution per plant can be quantified using the Gini index, which is linked to the Lorenz curve depicting the cumulative proportion of leaf (or fruit) size against the cumulative proportion of the number of leaves (or fruits). Prior researches have predominantly employed empirical models—specifically the original performance equation (PE‐1) and its generalized counterpart (GPE‐1)—to fit rotated and right‐shifted Lorenz curves. Notably, another potential performance equation (PE‐2), capable of generating similar curves to PE‐1, has been overlooked and not systematically compared with PE‐1 and GPE‐1. Furthermore, PE‐2 has been extended into a generalized version (GPE‐2). In the present study, we conducted a comparative analysis of these four performance equations, evaluating their applicability in describing Lorenz curves related to plant organ (leaf and fruit) size. Leaf area was measured on 240 culms of dwarf bamboo (*Shibataea chinensis* Nakai), and fruit volume was measured on 31 field muskmelon plants (*Cucumis melo* L. var. *agrestis* Naud.). Across both datasets, the root‐mean‐square errors of all four performance models were consistently smaller than 0.05. Paired *t*‐tests indicated that GPE‐1 exhibited the lowest root‐mean‐square error and Akaike information criterion value among the four performance equations. However, PE‐2 gave the best close‐to‐linear behavior based on relative curvature measures. This study presents a valuable tool for assessing the inequality of plant organ size distribution.

## Graphical abstract

Gini index can gauge the inequality of the size distribution of biological features. Lorenz curve calculating Gini index can be well described by performance equations. Four performance equations were compared using Lorenz curves of leaf and fruit size.

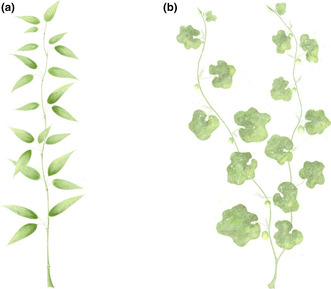

## 1 INTRODUCTION

Leaves and fruits play pivotal roles in the growth and development of seed plants. Foliage leaves, the primary photosynthetic organs in most vascular land plants (Wright et al., 2004), are instrumental in converting solar irradiance into chemical energy through photosynthesis (Rascher & Nedbal, 2006). Meanwhile, fruits, particularly fleshy ones, play a key role in seed dispersal by providing nourishment for various frugivorous animals (Willson, 1994). Literature has consistently emphasized the importance of leaf and fruit size, including parameters such as area, volume, and mass, in analyzing scaling relationships between these sizes (Guo et al., 2021, 2022; Li, Shi, et al., 2022; Milla & Reich, 2007; Niklas & Christianson, 2011; Pan et al., 2013; Shi, Miao, et al., 2022; Sun et al., 2017; Wang et al., 2023). Moreover, several mathematical models have been proposed and refined to describe leaf or fruit shape (Dornbusch et al., 2011; Gielis, 2003; Li, Niklas, et al., 2022; Li, Quinn, et al., 2022; Li, Zheng, et al., 2022; Lin et al., 2016; Schrader et al., 2021; Shi et al., 2015, 2020, 2021; Wang et al., 2023). These models offer a potential avenue for gaining novel insights into nondestructive estimation of size parameters such as leaf area, fruit surface area, and fruit volume (Lin et al., 2016; Wang et al., 2023; Yu et al., 2020). Despite this progress, the distribution of leaf or fruit size has been relatively understudied, representing an avenue ripe for further exploration.

**FIGURE 1:**
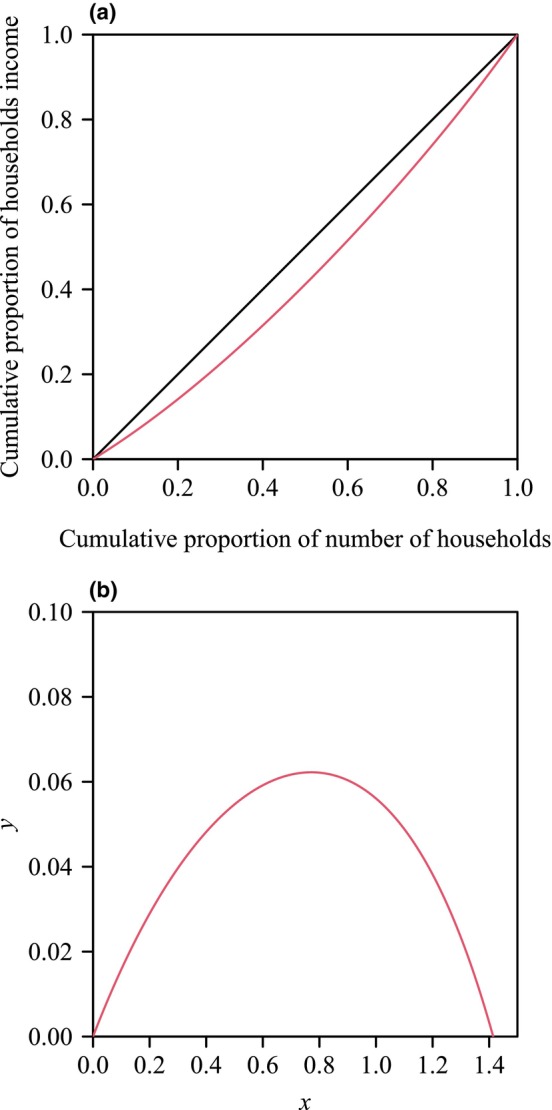
The Lorenz curve (depicted in red in panel a) and its rotated and right‐shifted version (shown in red in panel b) illustrate household income. In panel a, the black straight line represents the assumed line of absolute equality. In panel b, the Gini index is equal to double the integral of a performance curve (i.e., the rotated and right‐shifted Lorenz curve) from 0 to $\sqrt{2}$.

The Lorenz curve, originally applied to assess household income inequality (Gastwirth, 1971; Lorenz, 1905), depicts the cumulative proportion of household income against the cumulative proportion of the number of households (Figure 1a). In its extreme form, the Lorenz curve is a straight line passing through (0, 0) and (1, 1), representing absolute equality in household income distribution. Deviation from this line toward the point (1, 0) indicates income distribution inequality. The Gini index quantifies the degree of household income inequality by calculating the ratio of the area between the absolute equality straight line and the Lorenz curve to the area of a triangle formed by the points (0, 0), (1, 0), and (1, 1) (see Figure 1a). With values ranging from 0 to 1, a Gini index of 0 signifies absolute equality, while 1 indicates absolute inequality. Higher Gini indices denote greater degrees of inequality. Over recent decades, the Gini index has found broad applications in botany (Chen et al., 2014; Hara, 1986; Huang et al., 2023; Lian et al., 2023; Metsaranta & Lieffers, 2008; Taylor & Aarssen, 1989). In a recent study, Lian et al. (2023) introduced a unique approach by rotating the Lorenz curve counterclockwise by 135° around the origin and shifting it to the right by a distance of $\sqrt{2}$ (Figure 1b). This transformation was applied to explore the validity of a performance equation proposed by Huey (1975) and Huey and Stevenson (1979), denoted as PE‐1, in describing the rotated and right‐shifted Lorenz curve of leaf area and leaf dry mass distributions. The study by Lian et al. (2023) represents a novel application of established principles to botany, offering valuable insights into the analysis of plant organ size distributions.

While the validity of PE‐1 has been demonstrated in depicting the rotated and right‐shifted Lorenz curve of leaf size distribution (Lian et al., 2023), its applicability to describing the size distribution of other plant organs, such as fruits, remains uncertain. Furthermore, various mathematical equations have been proposed to generate skewed temperature‐dependent performance curves that bear a striking resemblance to the rotated Lorenz curve (Brière et al., 1999; Huey & Stevenson, 1979; Jin et al., 2022; Lobry et al., 1991; Ratkowsky et al., 1983; Shi et al., 2011, 2017). Consequently, there is a notable absence of studies applying different performance equations to assess the inequality of fruit size distribution. A comparative analysis of the effectiveness of various performance equations in describing the rotated and right‐shifted Lorenz curves of leaf or fruit size distribution is essential to fill this knowledge gap.

In this study, we digitized all leaves of 240 culms of *Shibataea chinensis* Nakai, with each culm having 11 to 35 leaves, to determine the area of each leaf. Additionally, we measured the volume of all fruits of 31 plants of *Cucumis melo* L. var. *agrestis* Naud., with each plant bearing 8 to 54 fruits. We employed four performance equations to fit the rotated and right‐shifted Lorenz curves using empirical data from the two species. Specifically, we focused on the cumulative proportion of leaf area versus the cumulative proportion of the number of leaves for *S. chinensis* and the cumulative proportion of fruit volume versus the cumulative proportion of the number of fruits for *C. melo* var. *agrestis*. The objectives were twofold: (i) to assess the accuracy of these performance equations in fitting the rotated and right‐shifted Lorenz curves of leaf and fruit size distribution; (ii) to identify which among the four performance equations provides the most accurate description of the inequality in size distribution of plant organs.

## 2 MATERIALS AND METHODS

### 2.1 Leaf and fruit sampling

**FIGURE 2:**
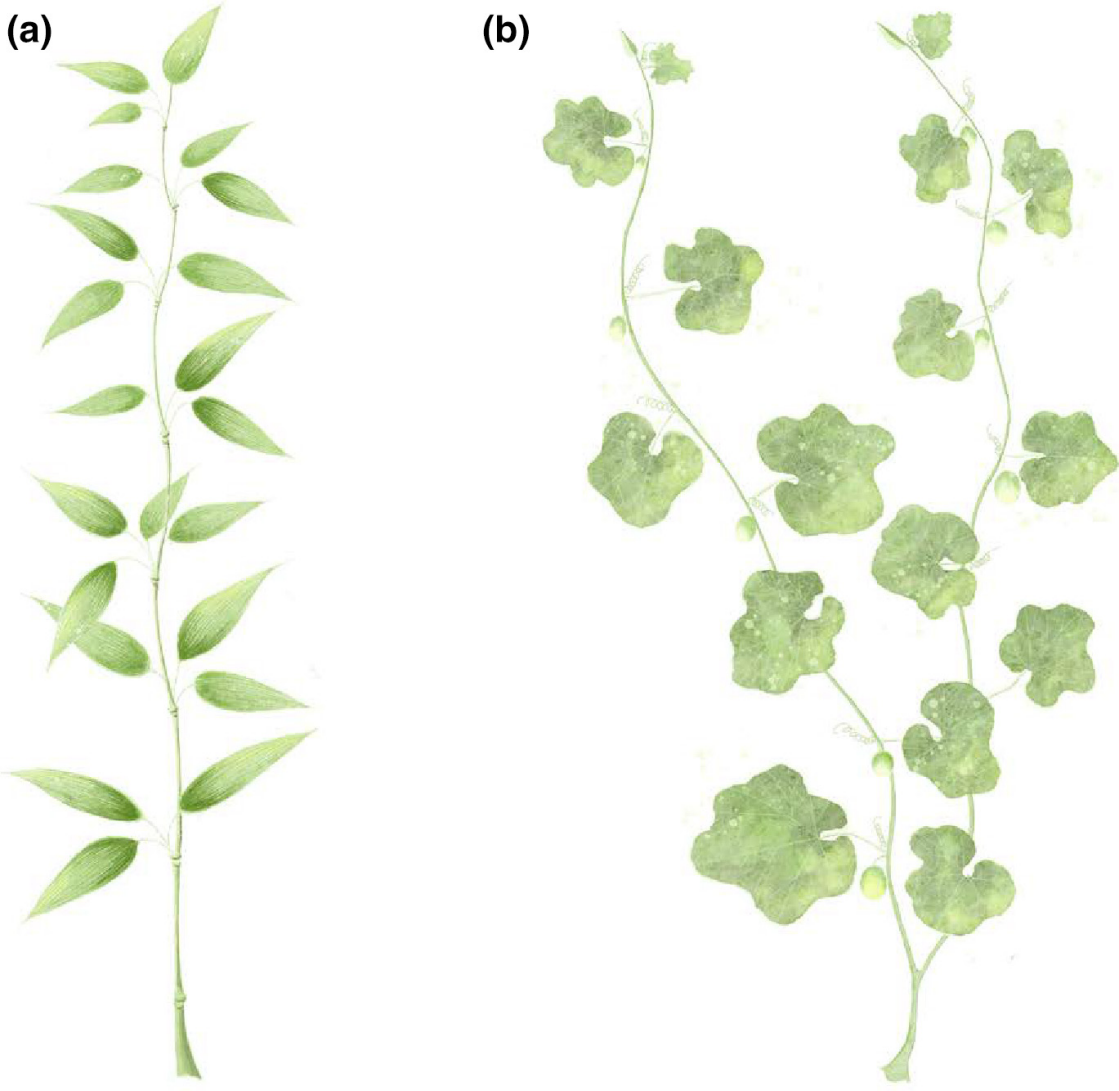
Freehand drawings of the aboveground part of (a) *Shibataea chinensis* and (b) *Cucumis melo* var. *agrestis*.

In early October 2022, we randomly sampled 240 individual culms of *S. chinensis* on the Nanjing Forestry University campus in Nanjing, Jiangsu Province, China (118°48′53″ E, 32°4′52″ N). To minimize water loss, each culm was promptly wrapped in wet paper after severing the culm at the ground and transported to the laboratory within 1 h. In the laboratory, leaves were carefully removed from each individual culm, and pseudo‐petioles were subsequently eliminated. Similarly, in early August 2023, we sampled 31 individual plants of *C. melo* var. *agrestis* in Changgou Town, Sixian, Anhui Province, China (117°44′26″ E, 33°29′14″ N). The vines of each plant were severed at the ground, placed in storage boxes, and brought to the laboratory within an hour. Fruits were then cut from each individual plant for subsequent measurement. Figure 2 provides a visual representation of the above‐ground architectural structures of both *S. chinensis* and *C. melo* var. *agrestis*.

### 2.2 Data acquisition

The laminas of *S. chinensis* were scanned at 600 dpi resolution using a photo scanner (V550, Epson, Batam, Indonesia) and saved as JPG files. Subsequently, Adobe Photoshop CS2 (version 9.0; Adobe, San Jose, CA, USA) was employed to convert the JPG images into black‐and‐white images saved as BMP files for each lamina. The specialized Matlab code (version ≥ 2009a; MathWorks, Natick, MA, USA) developed by Shi et al. (2015, 2018), was then used to extract the planar boundary coordinates of each lamina. The area of each leaf was calculated using the “bilat” function in the “biogeom” package (version 1.4.1; Shi, Gielis, et al., 2022) based on R (version 4.2.1; R Core Team, 2022). Additionally, the volume of *C. melo* var. *agrestis* fruits was measured by submerging each fruit in water in a 100 mL graduated cylinder with a 3 cm diameter and reading the observed volume of displaced water. The raw data for leaf area and fruit volume are accessible on the websites shown in the Data availability.

### 2.3 Models

The cumulative proportion of leaf area (or fruit volume) versus the cumulative proportion of the number of leaves (or fruits) underwent a counterclockwise rotation of 135° around the origin point and was subsequently shifted to the right by a distance of $\sqrt{2}$. The ensuing analysis employed four performance equations to model the rotated and right‐shifted data.

- The original performance equation, designated as PE‐1, was initially proposed to quantify the impact of temperature on the performance parameters of ectotherms (Huey, 1975; Huey & Stevenson, 1979; Shi et al., 2011):

(1) $$y=c\left(1-e^{-K_{1}\left(x-x_{1}\right)}\right)\left(1-e^{K_{2}\left(x-x_{2}\right)}\right),$$

where (*x*, *y*) corresponds to an arbitrary coordinate point after rotating and right‐shifting the original Lorenz curve, and *c*, *K*_1_, *K*_2_, *x*_1_, and *x*_2_ are parameters to be estimated.

ii To enhance the flexibility of empirical data fitting for leaves and fruits, Equation (1) can be extended to a generalized version by introducing two additional parameters (Lian et al., 2023). This generalized version is denoted as GPE‐1:

(2) $$y=c{\left(1-e^{-K_{1}\left(x-x_{1}\right)}\right)}^{a}{\left(1-e^{K_{2}\left(x-x_{2}\right)}\right)}^{b}.$$

iii Ratkowsky et al. (1983) proposed an empirical nonlinear regression model primarily designed to describe the temperature‐dependent growth rate of microorganisms, where the square root of the response variable produced a skewed bell‐shaped curve similar to that produced by Equation (1). Thus, the square root of the response variable was dropped to have the following formula:

(3) $$y=c\left(x-x_{1}\right)\left(1-e^{K_{2}\left(x-x_{2}\right)}\right).$$

This equation produces a skewed bell‐shaped curve similar to the rotated and right‐shifted Lorenz curve. Compared with Equations (1 and 3), has fewer parameters and a simpler model structure. We denoted Equation (3) as PE‐2.

iv In a similar vein, we introduced two additional parameters to Equation (3) to enhance the flexibility of the curve fitting, resulting in a generalized version denoted as GPE‐2:

(4) $$y=c{\left(x‐x_{1}\right)}^{a}{\left(1‐e^{K_{2}\left(x‐x_{2}\right)}\right)}^{b}$$

We can find that Equation (3) and the original square root model proposed by Ratkowsky et al. (1983) can be regarded as two special cases of Equation (4), that is, when *a* = *b* = 1 and *a* = *b* = 2 regardless of the parameter *c*.

The parameters *x*_1_, *x*_2_ in Equations ((1), (2), (3), (4)) represent the abscissas of the left and right intersections of the performance curve with the *x*‐axis, respectively. Notably, in the four performance equations mentioned above, we have *x*_1_ = 0 and *x*_2_ = $\sqrt{2}$.

### 2.4 Data fitting and model evaluation

We employed the four performance models described above (Section 2.3), namely PE‐1, PE‐2, GPE‐1, and GPE‐2, to fit rotated and right‐shifted Lorenz curves based on empirical data. This involved examining the cumulative proportion of leaf area versus the cumulative proportion of the number of leaves, and the cumulative proportion of fruit volume versus the cumulative proportion of the number of fruits. The Nelder–Mead optimization algorithm (Nelder & Mead, 1965) within a general‐purpose framework was utilized to minimize the fitting criterion of nonlinear regression. Parameters of the performance equations were estimated by minimizing the residual sum of squares (RSS) between empirical and predicted *y*‐values.

To evaluate the goodness of fit of the nonlinear regression, we calculated the root‐mean‐square error (RMSE) using the formula:

(5) $$\text{RMSE}=\sqrt{\mathrm{RSS}/\left(n-p\right)}$$

where *n* represents the number of leaves per culm (or fruits per plant), and *p* represents the number of parameters for each performance model. A smaller RMSE value indicates a better fit of the model. Additionally, we computed the Akaike information criterion (AIC) to balance the trade‐off between goodness of fit and model structural complexity (Burnham & Anderson, 2004). The model with the lowest AIC value is considered best. Paired *t*‐tests were employed to determine significant differences between RMSE or AIC values derived from different performance models.

In nonlinear analysis, it is crucial to evaluate the model's fit to the data and assess the appropriateness of assumptions related to disturbances and linear approximation (Bates & Watts, 1980, 1988). Bates and Watts (1988) assumed that the stochastic part, a disturbance perturbing the response in a nonlinear regression model, follows a spherical normal distribution. Furthermore, a local linear approximation, performed using the first‐order Taylor expansion at a fixed point in the model, underlies most algorithms for computing least squares estimates and most inference methods for nonlinear models. This involves two distinct assumptions: the planar assumption and the uniform coordinate assumption (Bates & Watts, 1980, 1988). Geometrically, the planar assumption implies that the expectation surface, also known as the solution locus (Box & Lucas, 1959), is approximated by the tangent plane. The uniform coordinate assumption means imposing a linear coordinate system on the approximating tangent plane. The adequacy of these two assumptions can be assessed through curvature measures of nonlinearity, categorized into intrinsic curvature and parameter‐effects curvature (Bates & Watts, 1980). Intrinsic curvature is an inherent property of the surface and depends solely on the mathematical model, remaining unchanged by model reparameterization. Conversely, parameter‐effects curvature relies on the chosen parameterization, and a nonlinear reparameterization of the model can significantly alter the parameter‐effects curvature (Bates & Watts, 1980; Lipschutz, 1969).

Although intrinsic curvature and parameter‐effects curvature serve as indicators of nonlinearity, they have limited utility due to their dependence on the data's scaling. To address this limitation, Bates and Watts (1980) introduced relative intrinsic curvature and relative parameter‐effects curvature, denoted as IC and PEC, respectively. These measures are independent of data scaling. Additionally, the root‐mean‐square curvature ($\gamma_{\mathrm{RMS}}$), defined as the square root of the average squared curvature over all directions, is commonly used to assess the quality of a linear approximation (Bates & Watts, 1988). The root‐mean‐square relative intrinsic curvature ($\gamma_{\mathrm{RMS}}^{N}$) is given by the equation:

(6) $$\gamma_{\mathrm{RMS}}^{N}=\sqrt{\frac{1}{A\left(p\right)}\int_{\left\|u\right\|=1}{\left({\mathrm{IC}}_{u}\right)}^{2}\mathrm{dA}}.$$

Similarly, the root‐mean‐square relative parameter‐effects curvature ($\gamma_{\mathrm{RMS}}^{T}$) is defined as:

(7) $$\gamma_{\mathrm{RMS}}^{T}=\sqrt{\frac{1}{A\left(p\right)}\int_{\left\|u\right\|=1}{\left({\mathrm{PEC}}_{u}\right)}^{2}\mathrm{dA}}.$$

Here, IC_*u*_ and PEC_*u*_ represent the relative intrinsic curvature and relative parameter‐effects curvature corresponding to the unit direction *u* in Equations (6 and 7), respectively (thus, ||*u*|| = 1). The surface area of the *p*‐dimensional unit sphere, denoted as *A*(*p*), depends on the number of parameters to be estimated in the nonlinear models (i.e., the performance models PE‐1, PE‐2, GPE‐1, and GPE‐2 in this context).

While root‐mean‐square curvatures provide a measure of nonlinearity, their interpretation may be challenging without a reference for what constitutes a “large” value. To address this, a critical curvature (*K*_*c*_) is established for comparison with $\gamma_{\mathrm{RMS}}^{N}$ and $\gamma_{\mathrm{RMS}}^{T}$ to assess the acceptability of the planar assumption and the uniform coordinate assumption associated with the linear approximation. *K*_*c*_ is defined as (Bates & Watts, 1980, 1988):

(8) $$K_{c}=\frac{1}{\sqrt{F\left(p,n-p;\alpha \right)}}.$$

Here, *F* represents the *F*‐distribution, *p* is the number of model parameters, *n* is the sample size (i.e., the number of leaves per culm or fruits per plant in this context), and α is the significance level typically set at 0.05. The value of $\sqrt{F\left(p,n-p;\alpha \right)}$ is considered the radius of curvature of the 100 (1−*α*)% confidence interval (Bates & Watts, 1980, 1988). If $\gamma_{\mathrm{RMS}}^{N}$ is smaller than *K*_*c*_, it suggests that the planar assumption is acceptable. Similarly, if $\gamma_{\mathrm{RMS}}^{T}$ is smaller than *K*_*c*_, the uniform coordinate assumption holds within the region of interest.

While $\gamma_{\mathrm{RMS}}^{T}$ offers a comprehensive measure of the overall nonlinearity behavior of all parameters in each model, it lacks the ability to provide insights into individual parameter performance. To assess the quality of the nonlinear fit for a specific parameter within a performance model, the percentage bias (*P*_*b*_) of each parameter can be employed, as suggested by Box (1971) and Ratkowsky (1983). For a specific parameter θ, the percentage bias (*P*_*b*_) is defined as:

(9) $$P_{b}=\frac{E\left(\hat{\theta }-\theta \right)}{\hat{\theta }}\times 100\%.$$

Here, θ represents the parameter to be estimated, $\hat{\theta }$ is the estimated value of θ, and *E* represents the expectation. As a general guideline, an absolute value of *P*_*b*_ <1% indicates that the nonlinear regression model is close‐to‐linear. This implies that the estimators of parameters exhibit several asymptotic properties, including proximity to unbiasedness, normal distribution, and minimization of variance (Ratkowsky, 1990).

Furthermore, the standardized skewness (*S*_*k*_) of the estimators of the parameters within a given performance model, equal to the standardized third moment of the estimators, can also be employed to evaluate the quality of the nonlinear fit for a specific parameter (Hougaard, 1985; Ratkowsky, 1990). Ratkowsky (1990) suggests a rule of thumb: an individual parameter demonstrates reasonably close‐to‐linear behavior if the corresponding absolute value of *S*_*k*_ is smaller than 0.25, although Haines et al. (2004) prefers a slightly more conservative value.

### 2.5 Quantifying the inequality of size distribution

The best performance model was selected for determining the Gini index:

(10) $$\text{Gini index}=2\times \int_{0}^{\sqrt{2}}\mathrm{ydx}$$

where *y* is defined by each of Equations ((1), (2), (3), (4)). The function “fitLorenz” in the “biogeom” package (version 1.4.1; Shi, Gielis, et al., 2022) based on R (version 4.2.1; R Core Team, 2022) was used to fit the rotated and right‐shifted Lorenz curves. The R package “IPEC” (version 1.1.0; Shi et al., 2024), built on R (version 4.2.1; R Core Team, 2022), was utilized for conducting nonlinear regression, parameter estimation, and calculations of all indicators related to nonlinearity.

## 3 RESULTS

**FIGURE 3:**
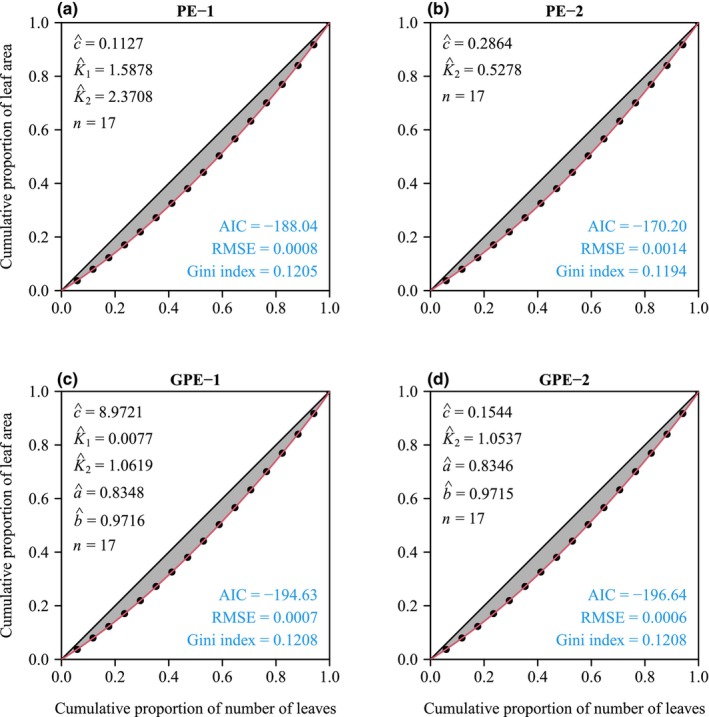
Comparison of the observed and predicted data of the original Lorenz curve for a representative individual culm of *Shibataea chinensis*. Data points represent observations; red curves signify predicted values. Letters *c*, *K*_1_, *K*_2_, *a*, and *b* with hats represent the estimated values of parameters of the corresponding performance equation in each panel; *n* represents the number of leaves on this individual culm; AIC represents the Akaike information criterion of the corresponding performance equation in each panel; RMSE represents the root‐mean‐square error. The Gini index calculated using the corresponding performance equation is provided in each panel.

**FIGURE 4:**
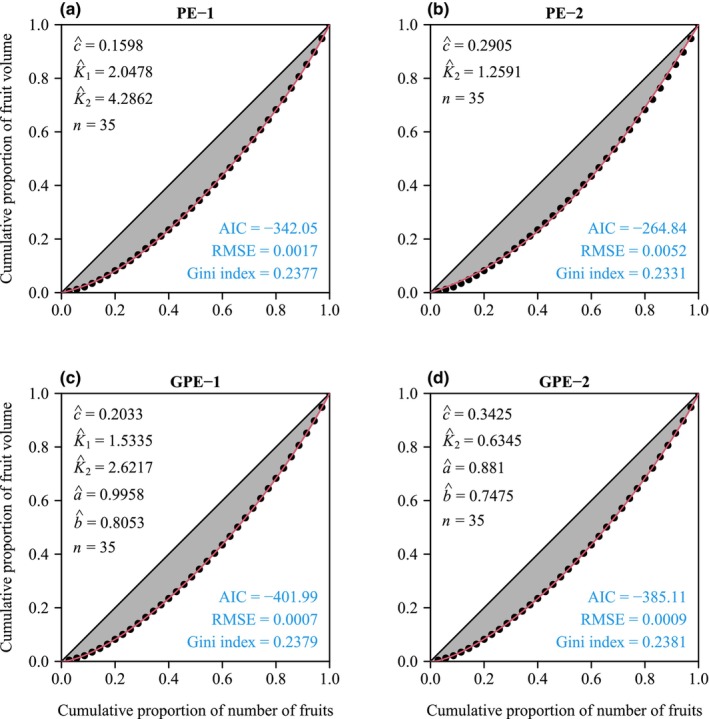
Comparison of the observed and predicted data of the original Lorenz curve for a representative individual plant of *Cucumis melo* var. *agrestis*. Data points represent observations; red curves signify predicted values. Letters *c*, *K*_1_, *K*_2_, *a*, and *b* with hats represent the estimated values of parameters of the corresponding performance equation in each panel; *n* represents the number of fruits on this individual plant; AIC represents the Akaike information criterion of the corresponding performance equation in each panel; RMSE represents the root‐mean‐square error. The Gini index calculated using the corresponding performance equation is provided in each panel.

**FIGURE 5:**
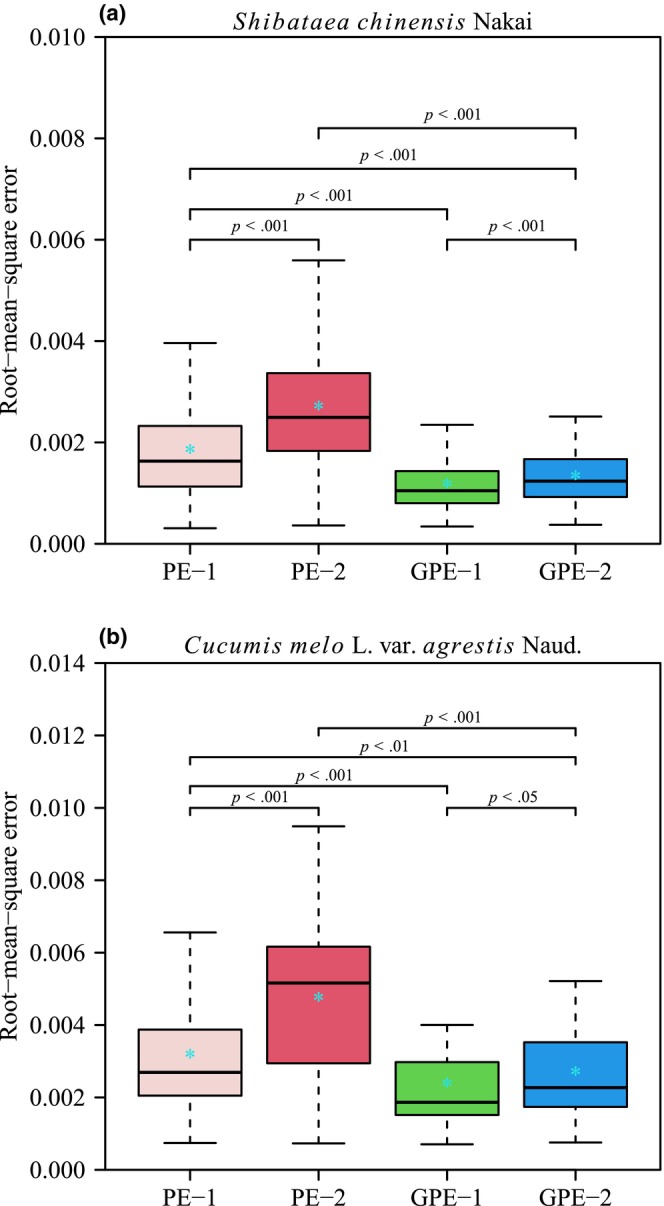
Boxplot of the root‐mean‐square error compared between the four performance equations (PE‐1, PE‐2, GPE‐1, and GPE‐2) for two datasets. Paired *t*‐test was used to determine the significant difference at the 0.05 significance level in the goodness of fit between any two of the four performance models. The vertical solid line in each box represents the median; the asterisk within the box represents the mean, and *p* represents the *p*‐value of the paired *t*‐test.

The four performance models (PE‐1, PE‐2, GPE‐1, and GPE‐2) generally provided effective representations of the rotated and right‐shifted Lorenz curves for both species. Figures 3 and 4 illustrate the fitting of original Lorenz curves for leaf area and fruit volume, respectively. For both species, the RMSE values for all four performance models were consistently below 0.05 (see Figure 5, Tables S1–S8). Paired *t*‐test results, conducted at a significance level of 0.05, indicated that GPE‐1 exhibited significantly lower RMSE values than the other three performance models for both species (see Figure 5, Table S9).

**FIGURE 6:**
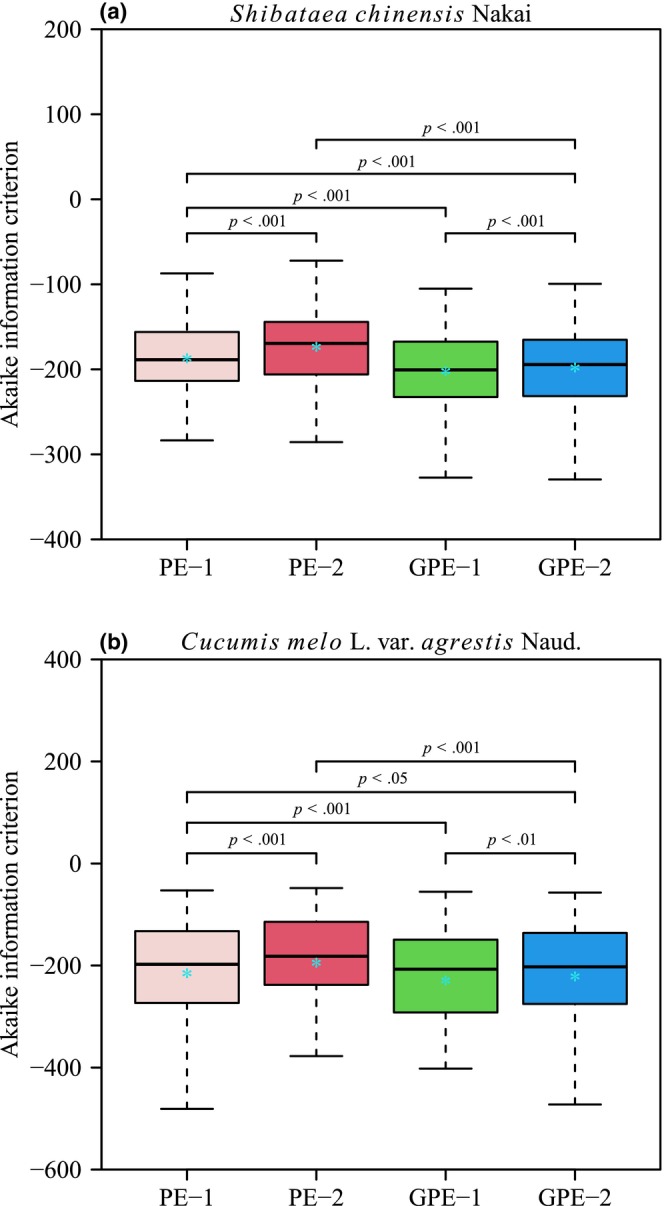
Boxplot of the Akaike information criterions compared between the four performance equations (PE‐1, PE‐2, GPE‐1, and GPE‐2) for two datasets. Paired *t*‐test was used to determine the significant difference at the 0.05 significance level in the AIC values between any two of the four performance models. The vertical solid line in each box represents the median; the asterisk within the box represents the mean, and *p* represents the *p*‐value of the paired *t*‐test.

These findings suggest that all four performance models effectively captured the characteristics of rotated and right‐shifted Lorenz curves for leaf area distribution in *S. chinensis* and fruit volume distribution in *C. melo* var. *agrestis*. Notably, GPE‐1 demonstrated the best goodness of fit for both species. Furthermore, the paired *t*‐test revealed that GPE‐1 had the lowest AIC values among the four performance models for the empirical data of both species, at a significance level of 0.05 (Figure 6, Table S10). This indicates that GPE‐1 outperformed the other models in nonlinear regression by achieving a favorable trade‐off between model structural complexity and goodness of fit.

**FIGURE 7:**
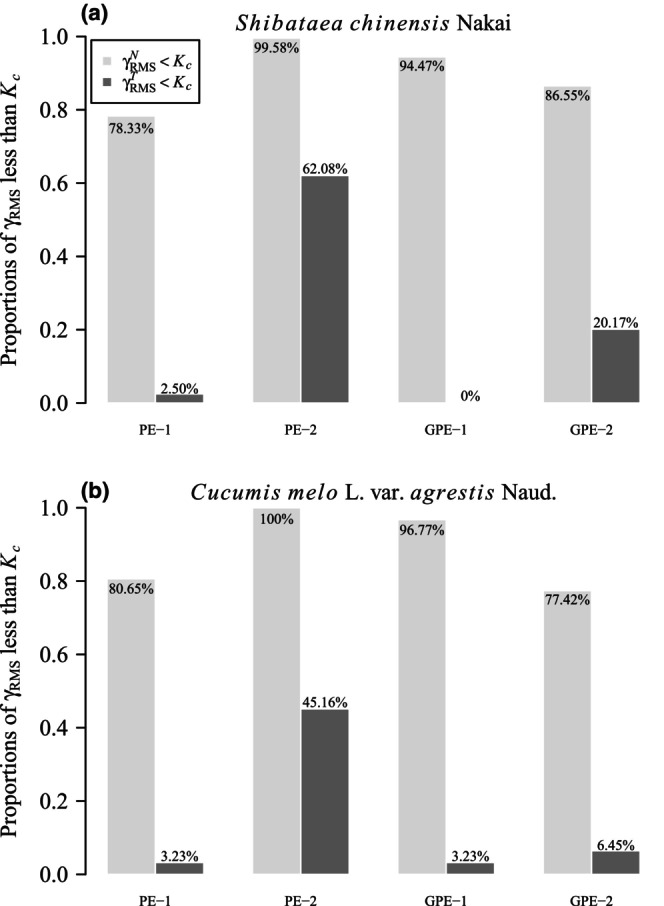
Bar chart of the comparison of root‐mean‐square relative curvature ($\gamma_{\mathrm{RMS}}$) with the critical curvature (*K*_*c*_) of the four performance equations (PE‐1, PE‐2, GPE‐1, and GPE‐2) for two datasets. $\gamma_{\mathrm{RMS}}^{N}$ represents root‐mean‐square relative intrinsic curvature; $\gamma_{\mathrm{RMS}}^{T}$ represents root‐mean‐square relative parameter‐effects curvature. For example, 78.33% in panel (a) represents for the performance equation PE‐1, there are 78.33% of 240 $\gamma_{\mathrm{RMS}}^{N}$ values that are smaller than the corresponding *K*_*c*_ for *Shibataea chinensis*; 45.16% in panel (b) represents for PE‐2, there are 45.16% of 31 $\gamma_{\mathrm{RMS}}^{T}$ values that are smaller than the corresponding *K*_*c*_ for *Cucumis melo* var. *agrestis*.

The nonlinearity of the performance models was assessed through overall relative curvature measures, namely $\gamma_{\mathrm{RMS}}^{N}$, $\gamma_{\mathrm{RMS}}^{T}$, and *K*_*c*_. For *S. chinensis* leaf data across the four performance models, 78.33% (PE‐1), 99.58% (PE‐2), 94.47% (GPE‐1), and 86.55% (GPE‐2) of the 240 culms had $\gamma_{\mathrm{RMS}}^{N}$ values smaller than the corresponding *K*_*c*_. Additionally, the proportions of $\gamma_{\mathrm{RMS}}^{T}$ values less than the corresponding *K*_*c*_ were 2.50% (PE‐1), 62.08% (PE‐2), 0% (GPE‐1), and 20.17% (GPE‐2) (Figure 7a, Tables S1–S4). For *C. melo* var. *agrestis* fruit data, across the four performance models, 80.65% (PE‐1), 100% (PE‐2), 96.77% (GPE‐1), and 77.42% (GPE‐2) of the 31 plants had $\gamma_{\mathrm{RMS}}^{N}$ values smaller than the corresponding *K*_*c*_. The proportions of $\gamma_{\mathrm{RMS}}^{T}$ values less than the corresponding *K*_*c*_ were 3.23% (PE‐1), 45.16% (PE‐2), 3.23% (GPE‐1), and 6.45% (GPE‐2) (Figure 7b; Tables S5−S8).

These results highlight that PE‐2 exhibits the best linear approximation among the four performance models. Notably, GPE‐1 demonstrates exceptional adherence to the planar assumption, with over 94% of $\gamma_{\mathrm{RMS}}^{N}$ values being smaller than the corresponding *K*_*c*_ in both species, indicating a well‐approximated solution locus by a plane. However, GPE‐1 falls short in confirming the uniform coordinate assumption, as over 96% of $\gamma_{\mathrm{RMS}}^{T}$ values are greater than the corresponding *K*_*c*_ in both species.

**FIGURE 8:**
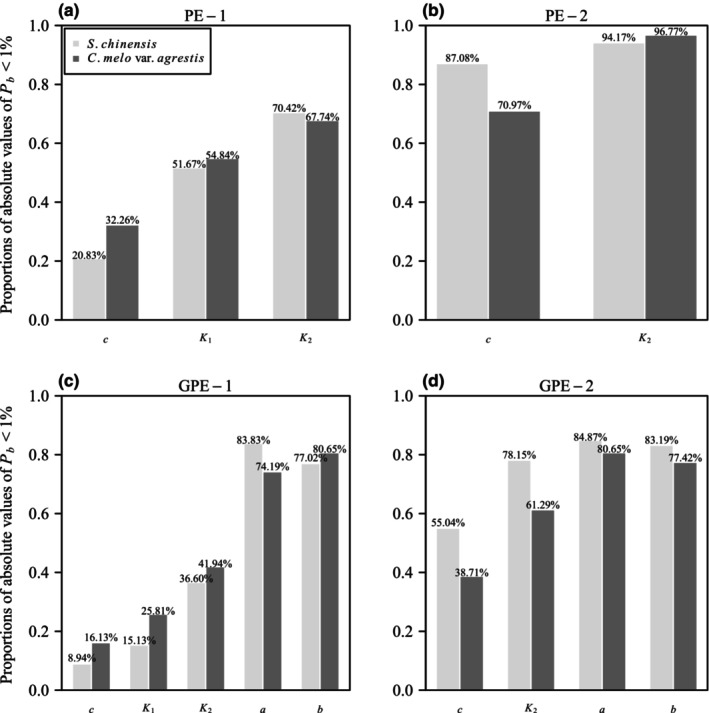
Bar chart of the proportions of the absolute values of percentage bias (*P*_*b*_) of each parameter in the four performance equations (PE‐1, PE‐2, GPE‐1, and GPE‐2) <1% for two datasets. For example, 20.83% in panel (a) represents there are 20.83% of the absolute values of *P*_*b*_ of parameter *c* of PE‐1, which are smaller than 1% for the data of *Shibataea chinensis*; 32.26% in panel (a) represents there are 32.26% of the absolute values of *P*_*b*_ of parameter *c* of PE‐1, which are smaller than 1% for the data of *Cucumis melo* var. *agrestis*. Panels (a–d) represent the four studied performance equations.

In terms of individual parameter‐level nonlinear behavior, examining the percentage bias (*P*_*b*_) of parameters reveals insightful findings. For PE‐1, the absolute values of *P*_*b*_ for *c*, *K*_1_, and *K*_2_ were smaller than 1% in 20.83, 51.67, and 70.42% of cases, respectively, in the data of *S. chinensis* leaves. Correspondingly, these proportions were 32.26, 54.84, and 67.74% for the data of *C. melo* var. *agrestis* fruits. Moving to PE‐2, 87.08 and 94.17% of the absolute values of *P*_*b*_ for *c* and *K*_2_ were below 1% for *S. chinensis*, and these figures were 70.97 and 96.77% for *C. melo* var. *agrestis*. For GPE‐1, 8.94, 15.13, 36.60, 83.83, and 77.02% of the absolute values of *P*_*b*_ for *c*, *K*_1_, *K*_2_, *a*, and *b*, respectively, were smaller than 1% in leaves data, and 16.13, 25.81, 41.94, 74.19, and 80.65% for fruits data. Regarding GPE‐2, 55.04, 78.15, 84.87, and 83.19% of the absolute values of *P*_*b*_ for *c*, *K*_2_, *a*, and *b*, respectively, were below 1% in leaves data, and 38.71, 61.29, 80.65, and 77.42% in fruits data (see Figure 8, Tables S1–S8).

These results underscore that PE‐2 exhibits the best close‐to‐linear behavior among the four performance models, with over 70% of the absolute values of *P*_*b*_ for each parameter being smaller than 1% in both species. GPE‐2 demonstrates relatively good close‐to‐linear behavior, except for parameter *c*, where <40% of the absolute values of *P*_*b*_ are <1%. PE‐1 exhibits moderate close‐to‐linear behavior (see Figure 8). However, GPE‐1 performs poorly, with three parameters (*c*, *K*_1_, and *K*_2_) having <42% of their absolute values of *P*_*b*_ below 1% for both datasets.

**FIGURE 9:**
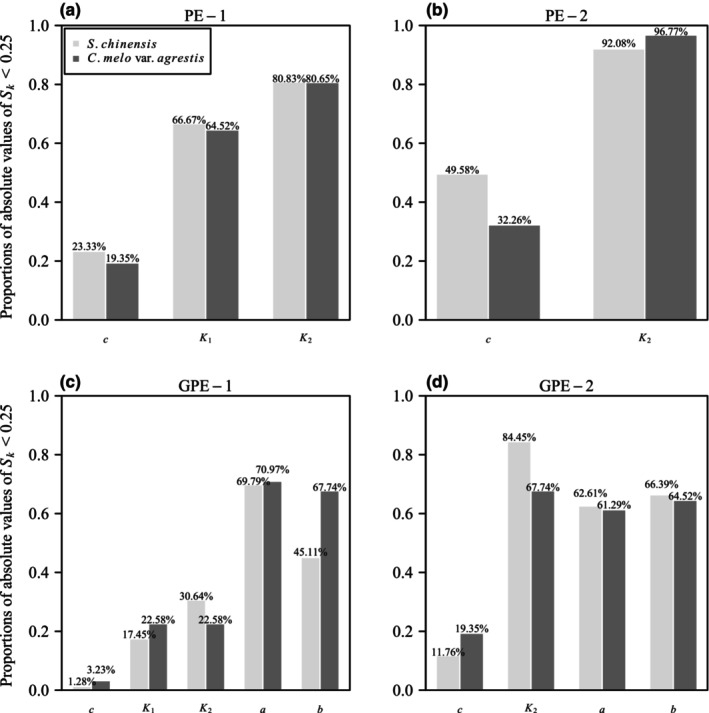
Bar chart showing the proportions of absolute values of standardized skewness (*S*_*k*_) for each parameter in the four performance equations (PE‐1, PE‐2, GPE‐1, and GPE‐2) that are <0.25 for two datasets. For instance, in panel (a), 23.33% represents the percentage of absolute *S*_*k*_ values of parameter *c* in PE‐1 that are smaller than 0.25 for the data of *Shibataea chinensis*; while in panel (a), 19.35% signifies the percentage of absolute *S*_*k*_ values of parameter *c* in PE‐1 that are smaller than 0.25 for the data of *Cucumis melo* var. *agrestis*. Panels (a–d) represent the four studied performance equations.

Regarding the standardized skewness (*S*_*k*_) of the parameters, the detailed results are presented in Figure 9, Tables S1–S8. PE‐2 exhibited the best close‐to‐linear behavior, with <8% of the absolute values of *S*_*k*_ for parameter *K*_2_ exceeding 0.25, and over 32% of the absolute values of *S*_*k*_ for parameter *c* being smaller than 0.25 in both species. For the remaining three equations, PE‐1 and GPE‐2 showed relatively better close‐to‐linear behavior, excluding parameter *c*, when compared to GPE‐1 (see Figure 9). GPE‐1 displayed unfavorable behavior across three parameters (*c*, *K*_1_, and *K*_2_), with <31% of their absolute values of *S*_*k*_ being smaller than 0.25 in both datasets.

We employed GPE‐1 to compute the Gini indices for both species, given its superior goodness of fit and the lowest AIC values. The Gini indices for the inequality measure of leaf area distribution in *S. chinensis* ranged between 0.046 and 0.255 (refer to Table S3). Similarly, for the inequality measure of fruit volume distribution in *C. melo* var. *agrestis*, the Gini indices ranged between 0.120 and 0.383 (refer to Table S7).

## 4 DISCUSSION

In assessing nonlinear regression models, the goodness of fit and the Akaike information criterion are commonly employed criteria. However, these may prove insufficient when comparing complex nonlinear models with numerous parameters (Ratkowsky & Reddy, 2017). Beyond fitting data, a robust nonlinear model should ensure that each parameter exhibits close‐to‐linear behavior, ensuring that their least squares estimators are nearly unbiased, normally distributed, and represent minimum variance estimators (Ratkowsky, 1990; Ratkowsky & Reddy, 2017). Consequently, the selection of an appropriate model based on these criteria is a pivotal step in any nonlinear analysis.

The four performance models under investigation can be categorized into two groups based on their goodness of fit. Notably, GPE‐1 and GPE‐2 emerge as models that effectively capture the observed data, as illustrated in Figure 5. Both GPE‐1 and GPE‐2 exhibit a robust performance in adhering to the planar assumption, with over 77% of $\gamma_{\mathrm{RMS}}^{N}$ values being smaller than the corresponding *K*_*c*_ in both species (Figure 7). Despite GPE‐1 displaying a strong fit to the empirical data, only two of its five parameters, namely *a* and *b*, demonstrate close‐to‐linear behavior, as assessed by metrics such as lower percentage bias and skewness (Figures 8 and 9). In contrast, three out of the four parameters of GPE‐2, i.e., *K*_2_, *a*, and *b*, consistently exhibit close‐to‐linear behavior across both datasets (Figures 8 and 9).

On the opposite end of the goodness‐of‐fit spectrum, PE‐1 and PE‐2 are found to be less adept at fitting the observed data (Figure 5). Despite their shortcomings in goodness of fit, these models demonstrate commendable performance in terms of being close‐to‐linear. For instance, PE‐2, despite having the poorest goodness of fit among the four models, boasts the best linear approximation (Figure 7). Furthermore, at the individual parameter level, all parameters of PE‐2, from *c* to *K*_2_, are deemed close‐to‐linear based on lower percentage bias and skewness (Figures 8 and 9).

After a comprehensive evaluation of the nonlinear regression models through various methodologies, it can be concluded that PE‐2 exhibited the poorest goodness of fit and the highest AIC values, whereas GPE‐1 showcased the most favorable goodness of fit with the lowest AIC values. However, despite PE‐2's suboptimal goodness of fit, it demonstrated the best close‐to‐linear behavior among the four performance models, both as an overall measure and at the individual parameter level of nonlinear behavior. On the other hand, GPE‐1 faced challenges related to the uniform coordinate assumption and displayed drawbacks in the behavior of five of its parameters, with three, *c*, *K*_1_, and *K*_2_, not demonstrating close‐to‐linear characteristics. Therefore, in the context of the four performance models examined, GPE‐1 stands out as the preferred choice, particularly when prioritizing goodness of fit and AIC. However, if the focus shifts to nonlinear behavior, PE‐2 might be considered the optimal selection. It is essential to note that future studies on different species may lead to different conclusions. The determination of the most suitable model ultimately lies in the hands of the researchers.

The parameter *c* in GPE‐1 exhibited a pronounced departure from linearity, with over 80% of the absolute values of the percentage bias exceeding 1% in both datasets, and <4% of the absolute values of skewness falling below 0.25 (Figures 8 and 9). However, nonlinear reparameterization has the potential to enhance equation nonlinearity, resulting in less biased and skewed least squares estimators (Ratkowsky & Reddy, 2017). Such a reparameterization could also reduce parameter‐effects curvature (Bates & Watts, 1980). In this study, we introduced a reparameterized version of GPE‐1, denoted as Repar‐GPE‐1, where *c* was replaced by the exponential of itself, that is,

(11) $$y=e^{c}{\left(1-e^{-K_{1}\left(x-x_{1}\right)}\right)}^{a}{\left(1-e^{K_{2}\left(x-x_{2}\right)}\right)}^{b}.$$

**FIGURE 10:**
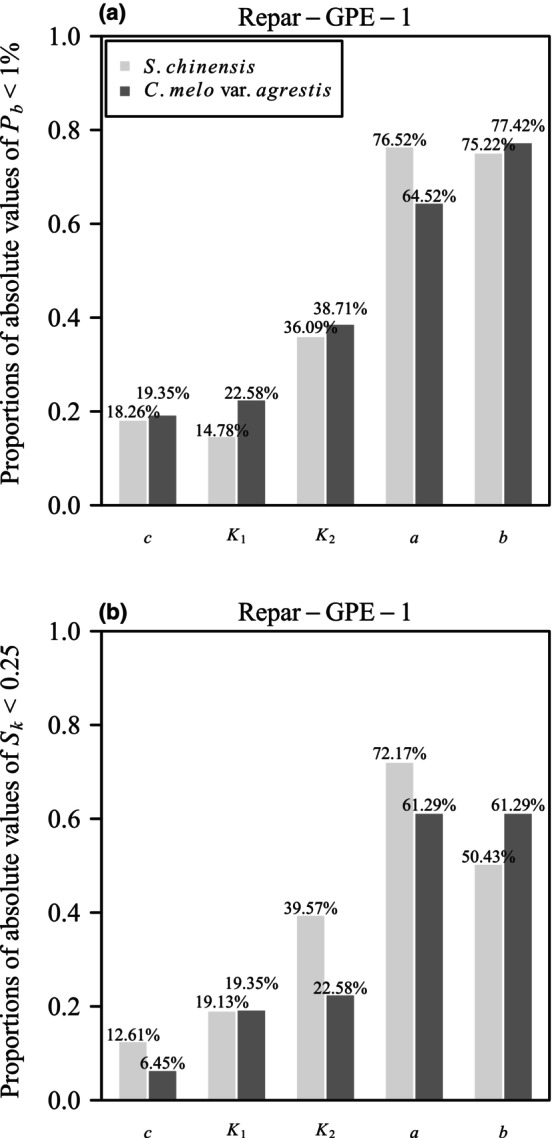
Nonlinearity measures for Repar‐GPE‐1. (a) Bar chart illustrating the proportions of absolute values of percentage bias (*P*_*b*_) for each parameter in the reparametrized performance equation Repar‐GPE‐1 that are <1% for two datasets. (b) Bar chart depicting the proportions of absolute values of standardized skewness (*S*_*k*_) for each parameter in the reparametrized performance equation Repar‐GPE‐1 that are <0.25 for two datasets.

The rotated and right‐shifted Lorenz curves of empirical data were then fitted using Repar‐GPE‐1. The results revealed a significant improvement in the close‐to‐linear behavior of the new parameter *c* in Repar‐GPE‐1 compared to GPE‐1. The proportions of absolute values of the percentage bias <1% increased from 8.94% to 18.26% for the *S. chinensis* dataset and from 16.13 to 19.35% for the *C. melo* var. *agrestis* dataset (Figures 8c and 10a, Tables S3, S7, S11, and S12). Additionally, the proportions of absolute values of the skewness measurement <0.25 increased from 1.28 to 12.61% for *S. chinensis* and from 3.23 to 6.45% for *C. melo* var. *agrestis* (Figures 9c and 10b, Tables S3, S7, S11, and S12).

However, it is important to note that identifying an appropriate reparameterization form to enhance a model is a challenging task for researchers, primarily because nonlinear reparameterization may lead to a more complex model structure. Exploring suitable reparameterization methods to improve performance models could be a valuable avenue for future studies, potentially enhancing the ability to describe the inequality of plant organ size distribution more accurately.

## 5 CONCLUSIONS

The application of four performance equations to fit rotated and right‐shifted Lorenz curves describing empirical data on plant organ size distribution yielded distinctive results. Specifically, PE‐2 demonstrated the best close‐to‐linear behavior among the models, yet it exhibited the poorest goodness of fit and the highest AIC value. Conversely, GPE‐1 displayed the best goodness of fit, characterized by the lowest AIC value, but yielded far‐from‐linear least‐squares estimates for parameters governing the rotated and right‐shifted Lorenz curves. Consequently, the choice of model for assessing inequality in leaf or fruit size distribution hinges on considerations of goodness of fit, model structural complexity, and the close‐to‐linear behavior of parameters. In situations where two models exhibit similar goodness of fit, evaluating the close‐to‐linear or far‐from‐linear behavior of parameters becomes pivotal in selecting the optimal model. This nuanced approach provides valuable insights into the criteria governing model selection for nonlinear regression in quantifying plant organ size distribution inequality.

## AUTHOR CONTRIBUTIONS

**Lin Wang:** Formal analysis (equal); methodology (equal); writing – original draft (equal). **Ke He:** Investigation (equal); writing – original draft (equal). **Cang Hui:** Formal analysis (equal); writing – review and editing (equal). **David A. Ratkowsky:** Methodology (equal); writing – review and editing (equal). **Weihao Yao:** Investigation (equal); writing – review and editing (equal). **Meng Lian:** Investigation (equal); writing – review and editing (equal). **Jinfeng Wang:** Investigation (equal); writing – review and editing (equal). **Peijian Shi:** Formal analysis (equal); methodology (equal); supervision (lead); writing – review and editing (equal).

## CONFLICT OF INTEREST STATEMENT

The authors declare that they have no known competing financial interests or personal relationships that could have appeared to influence the work reported in this paper.

## Supporting information


Tables S1–S12.

## Data Availability

The data underlying this article are available in the Dryad Digital Repository, at https://doi.org/10.5061/dryad.mgqnk995f, for the fruit volume distributions of 31 individuals of *Cucumis melo* var. *agrestis*, and in the supplementary materials of Huang et al. (2023), https://www.mdpi.com/article/10.3390/plants12173143/s1, for the leaf area distributions of 240 culms of *Shibataea chinensis*, and in the online supplementary material of this article for the results of data fitting (Tables S1–S12).
